# Infected Feather Beds

**Published:** 1895-05

**Authors:** 


### INFECTED FEATHER BEDS.

The most unsanitary of all household articles is the feather bed. Quite too frequently it is an heirloom which has come down through many generations past, and at times it proves to be a genuine Pandora's box of germs, and malodors and other unsanitary things which have accumulated during the several generations in which it has done service for all sorts of people under all sorts of conditions. In the larger cities, convenient renovating establishments afford facilities for the purification of feather beds, pillows, etc., which to some degree remedies the evil of which we complain, but by no means altogether; for the feather bed, at best, contains a considerable amount of organic matter clinging to the quills and feathers, which, absorbing the waste of the body, is always undergoing decomposition, throwing off poisonous gases into the air and affording food for myriads of pestilential microbes which are ever in readiness to seize a favorable opportunity of infecting a weakened body, setting up suppurating processes and intensifying the effects of specific germs of various sorts which may become active in the body through the contagion.

Sometimes, also, a feather bed becomes infected by the contagious elements of scarlet fever, diphtheria, measles, smallpox, or other maladies, and constitutes thereby a most efficient vehicle for these dangerous disorders.

A case of this sort recently occurred in an Eastern town. The death of two children in Woburn, Mass., led to an investigation of the infection, which disclosed the fact that two weeks before the illness of the children a barrel containing a feather bed had been dumped upon a vacant lot. A number of children playing in the lot next day discovered the feather bed, opened it, and scattered the feathers over one another, thus effecting the most thorough exposure possible to whatever contagious element the feathers might contain. Within a few days five of these children were taken with scarlet fever; two died shortly afterward, and at the time the case was reported the third child was not expected to live. After the children were taken ill someone, perhaps the originally guilty party, burned the feather bed.

In such a case the neglect to destroy so efficient an agent of dissemination of disease before giving opportunity for such a fatality as above reported, cannot be looked upon as anything less than criminal neglect and carelessness.

That this fact was appreciated seems to be evidenced by the subsequent burning of the bed after the children were taken sick. It is to be hoped that the guilty party was discovered, and proper legal punishment administered. It is no less a crime to destroy human life in such a manner than by neglect of proper care for a steam boiler, or by carelessly running a steamboat upon a rock, or, through neglect, allowing it to fire and burn up in mid ocean.—*Kansas Medical Journal*, March 9, 1895.

